# Draft Genome Sequence of *Lactobacillus gasseri* Strain 2016

**DOI:** 10.1128/genomeA.00624-13

**Published:** 2013-08-22

**Authors:** Andrey V. Karlyshev, Vyacheslav G. Melnikov, Igor V. Kosarev, Valentin C. Khlebnikov, Gennady T. Sukhikh, Vyacheslav M. Abramov

**Affiliations:** School of Life Sciences, Faculty of Science, Engineering and Computing, Kingston University, Kingston upon Thames, United Kingdoma; International Science and Technology Centre, Moscow, Russiab; Institute of Immunological Engineering, Lyubuchany, Moscow Region, Russiac; Research Center of Obstetrics, Gynecology and Neonatology, Ministry of Health and Social Development of the Russian Federation, Moscow, Russiad

## Abstract

Different common factors contribute to the antagonistic properties of *Lactobacillus gasseri* toward various pathogens. However, there is strain-to-strain variation in the probiotic properties of this bacterium. The draft genome sequence of *L. gasseri* strain 2016 determined in this study will assist in understanding the genetic basis for such variation.

## GENOME ANNOUNCEMENT

The emergence of multidrug-resistant bacterial pathogens and the high incidence of urogenital infections, particularly affecting women (hundreds of millions cases worldwide), necessitate the development of alternative antibacterial agents (1). *Lactobaccilus* spp. have been found to be very promising for the treatment of these (and other) illnesses (2).

Screening of 412 strains of *Lactobacillus gasseri* resulted in the isolation of a highly adhesive and bacteriocinogenic strain of *L. gasseri*, 2016, which produces high levels of hydrogen peroxide (our unpublished observations). This strain is deposited at the All-Russian Collection of Microorganisms at the GK Skryabin Institute of Biochemistry and Physiology of Microorganisms (Russian Academy of Sciences, Moscow Regions, Puschino) under the registration number VKM В**-**2728D. In this study, we determined a draft genome sequence of *L. gasseri* 2016 using IonTorrent PGM.

Sequencing on a 314 chip produced 371,671 reads with a total size of 42.2 million bases. Assembly of reads by use of the IonTorrent assembler plugin produced 440 contigs (0.5 to 24.2 kb; total size, 1,609,472 bases; GC content, 35.2%). At the time of preparation of materials for this publication, the only complete genome sequence of *Lactobacillus gasseri* available was that for strain ATCC 33323 (accession number NC_008530; genome size, 1,894,360 bp; GC content, 35.3%) reported in 2006 (3). Mapping of reads onto the genome of the reference strain by use of the CLC Genomics Workbench program allowed the generation of 10 consensus sequences with a size range from 1,302 to 795,849 bases. The unmapped reads were assembled onto 19 contigs between 1,048 and 11,426 bases in size. The combined size of mapped and unmapped contigs was 1,879,515 bases, corresponding to 99.2% of the reference genome. The contigs were verified by mapping the reads to contigs. The total number of reads mapped was 35,186,093 (94.67%), corresponding to 21.25-fold genome coverage.

Mapping of reads onto the reference genome revealed a large number of common genes, including a gene encoding helveticin (100% identity). Typical missing genes corresponded to integrases and recombinases related to various prophages as well as transposons. The assembled unmapped reads were further analyzed in order to identify the genes that are present in strain 2016 but were absent in the reference strain. These contigs were analyzed using a batch BLASTx utility of CLC Genomics Workbench and a nonredundant amino acid sequence database. Products of some genes revealed high levels of similarity to cell surface proteins found in other *Lactobacillus* spp., including a putative cell wall surface anchor family protein (*L. rhamnosus* LRHMDP2, 67% identity in 543 amino acids), cell surface protein (*L. antri* DSM 16041, 99% identity in 477 amino acids), and mannose-specific adhesin, LPXTG-motif cell wall anchor (*L. plantarum* WCFS1, 68% identity in 484 amino acids). The additional genes and features of the sequenced strain may reflect a different lifestyle of this strain and contribute to the beneficial effects of this strain as a probiotic.

### Nucleotide sequence accession number. 

The shotgun genome sequence of *L. gasseri* 2016 in a form of 29 contigs, including both mapped consensus sequences and unmapped contigs, was deposited at GenBank under the accession number AUUE00000000 (released 25 July 2013).
